# Novel characterization method of impedance cardiography signals using time-frequency distributions

**DOI:** 10.1007/s11517-017-1776-x

**Published:** 2018-03-16

**Authors:** Jesús Escrivá Muñoz, Y. Pan, S. Ge, E. W. Jensen, M. Vallverdú

**Affiliations:** 1 Biomedical Engineering Research Center, CIBER of Bioengineering, Biomaterials and Nanomedicine (CIBER-BBN), Universitat Politècnica de Catalunya, Barcelona, Spain; 2 Quantium Medical, SL, Barcelona, Spain; 3 Zhongshan Hospital, Shanghai, China

**Keywords:** Impedance cardiography, Time-frequency distributions

## Abstract

The purpose of this document is to describe a methodology to select the most adequate time-frequency distribution (TFD) kernel for the characterization of impedance cardiography signals (ICG). The predominant ICG beat was extracted from a patient and was synthetized using time-frequency variant Fourier approximations. These synthetized signals were used to optimize several TFD kernels according to a performance maximization. The optimized kernels were tested for noise resistance on a clinical database. The resulting optimized TFD kernels are presented with their performance calculated using newly proposed methods. The procedure explained in this work showcases a new method to select an appropriate kernel for ICG signals and compares the performance of different time-frequency kernels found in the literature for the case of ICG signals. We conclude that, for ICG signals, the performance (P) of the spectrogram with either Hanning or Hamming windows (*P* = 0.780) and the extended modified beta distribution (*P* = 0.765) provided similar results, higher than the rest of analyzed kernels.

**Graphical abstract:**
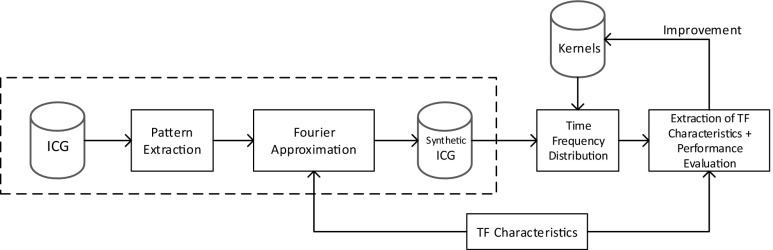
Flowchart for the optimization of time-frequency distribution kernels for impedance cardiography signals.

Flowchart for the optimization of time-frequency distribution kernels for impedance cardiography signals.

## Introduction

In patients undergoing surgery, scientific evidence supports the correlation of advanced hemodynamic monitoring with good tissue perfusion and better hemodynamic optimization. This improves patient outcome and reduces mortality rates and hospital costs [1–5]. However, monitoring of cardiovascular hemodynamic changes requires specialized equipment which can be very expensive. Impedance cardiography (ICG) is an inexpensive, non-invasive method which uses the fact that different human tissues are characterized by different impedances. While bones, lungs, and muscles are poor electrical conductors, blood offers low resistance to currents, and therefore, thoracic ICG signals represent the change in the cardiac blood volume.

Advanced techniques for the analysis of biomedical signals such as the ICG signals and automatization techniques are increasingly important for diagnosis. The visual inspection of biomedical signals may be a tedious task, and subjective judgements and errors can occur even when skilled interpreters are involved.

Several hemodynamic indices can be extracted from ICG signal [6, 7], and applications range from stroke volume calculation to diagnosis of cardiac or work-related conditions [8–10]. In order to improve the calculation of these indices, several authors have exploited the periodic or quasi-periodic behavior of the ICG signals for denoising or for locating their characteristic points [11, 12]. The study of these signals in the frequency domain can shed light on the quasi-periodical behavior of ICG signals and also on ICG features which cannot be directly observed in the time domain. Some features could be related to HRV, for which ECG analysis would be more effective, but other features could be related to the patient’s hemodynamic state. However, the ICG behavior in the frequency domain varies with time, and therefore, it is convenient to analyze how the frequency distribution of the signal changes with time [13].

Time-frequency distribution (TFD) studies how the frequency content changes with time, and it has been used for analyzing electroencephalogram (EEG) [14–18], evoked potentials [19], heart rate variability (HRV) [20–23], and pathological speech signals [24, 25], amongst others. There are several different ways to formulate valid TFDs [26], but any application would ideally require high definition in spectral components, no cross-terms (in order to avoid confusing real components from artifacts or noise), a low computational complexity, and some mathematical properties [27]. However, these properties do not normally occur together, and the selection of an appropriate TFD depends on the characteristics of the signal to analyze.

When it comes to selecting the best TFD for synthetic signals, several quantitative measurements can be used based on geometrical properties [28, 29], error, and entropy properties [30–33] of the TFDs. These measurements can be applied when the time-frequency characteristics of real signals are known. Nevertheless, these characteristics are usually unknown in real physiological signals.

In this work, in order to test the goodness of TFDs for real biological signals such as the ICG, we propose the usage of synthetic signals modeling the real ICG signals. The TFDs can then be optimized for its usage with real ICG signals by means of using ICG synthetic signals fulfilling the following requirements: synthetic signals should resemble very much to the original signals from a time-frequency perspectives, and time-frequency parameters should be easily modifiable. To the best of our knowledge, the application of TFDs to ICG signals has not been extensively researched.

In order to accomplish these requirements, a pattern analyzer was designed and used on a real ICG signal to decipher a patient’s typical ICG beats. This patient was randomly chosen from a database of patients scheduled for general anesthesia in the Zhongshan Hospital (Shanghai, PRC). The most frequent pattern was calculated and a Fourier series model was created. This Fourier model was modified in order to create signals with time-frequency variations. These synthetic signals were used to evaluate different TFDs based on geometric criteria. The most common TF distributions available were tested: the Wigner-Ville distribution (WVD), the Born-Jordan distribution (BJD), the spectrogram (SP), the S-Method (SM), the Choi-Williams distribution (CWD), the Zhao-Atlas-Marks distribution (ZAM), the modified B-distribution (MBD), and the extended modified B-distribution (EMBD) [26]. The instantaneous frequency (IF) of the different spectral components on the TFDs was determined, and its errors with theoretical IFs were measured. The robustness against low signal-to-noise ratios has also been tested. In addition, optimized TFDs were used on synthetic ICG signals derived from patterns of the rest of patients in the ICG database.

## Methods

### ICG database

ICG signals were obtained from a study conducted at the Zhongshan Hospital (Shanghai, People’s Republic of China) under the requirements of its Ethical Committee and the protocol adhered to the principles of the Declaration of Helsinki. ECG signals were concurrently acquired. The study included 15 patients undergoing major surgery in an observational study. Age ranged between 43 and 67 years, with a mean age of 58.9 ± 6.7 years and a mean body mass index of 22.8 ± 2.6 kg/m^2^. These signals were obtained using the qCO monitor (Quantium Medical, Barcelona, Spain). This monitor registers the impedance cardiography (ICG) and electrocardiogram (ECG) by using four electrodes, with one pair injecting a constant current of 50 kHz and a second pair of electrodes measuring the resulting voltage. The impedance signal represents the changes of the thoracic impedance due to variations in the blood flow.

In practice, the raw impedance signal is transformed into the negative time-derived impedance waveform by using the first derivative to remark the inflection points of the raw impedance signal. This signal is also low-pass filtered at 30 Hz to reduce high-frequency noise in both the forward and reverse directions to avoid zero-phase distortion. All the signal processing techniques have also been developed using MATLAB^®^.

### Synthetic ICG signal generated

Synthetic ICG signals were generated in order to select the best TFDs for the real ICG signals. Firstly, the most typical waveform of a real ICG recording was recognized. This pattern was later used to create a Fourier model approximation with several instantaneous frequencies (IFs), and synthetic signals were created including concrete variable time-frequency characteristics.

#### Pattern recognition analysis

A pattern recognition algorithm has been designed to detect the most typical waveforms which are contained in the ICG signals. Each ICG beat is isolated and normalized for zero mean and unit standard deviation. Moreover, the length of all ICG beats is normalized to a constant number of samples. The starting and endpoints are defined as the QRS peaks in the ECG before and after an ICG maximal peak. Moreover, ICG maxima were located using the QRS peaks in the ECG, which are easier to locate with a Pan-Tompkins approach [30].

The first pattern of the database is the first ICG beat available in the recording. The rest of ICG beats are correlated with all the patterns in the database. For each ICG beat, the pattern which offers a higher Pearson correlation above a threshold *th* (*th* > 0.85) with the beat is then averaged with such beat. If the correlation threshold is not met, a new pattern is created. Moreover, the algorithm ensures that there will not be two patterns with a cross-correlation higher than 0.95.

#### Fourier modeling

If a long periodic ICG signal has a most frequent pattern which repeats itself periodically every *N*_*p*_ samples with a frequency *ω*_*n*_ = 2*π*/*N*_*p*_, the ICG signal can be represented as a Fourier series. This Fourier series requires an infinite number of terms to accurately reproduce the square wave signal. Generally, the model for the pattern ICG signal will have a structure with a defined number of terms of a discrete-time Fourier series of frequency *ω*_*n*_ = 2*π*/*N*_*p*_ where *N*_*p*_ will be the length of the single pattern.

The Fourier model for the template signal allows modifying the time-frequency characteristics of a longer signal *x*(*n*) in a controlled, straightforward fashion, similar to the time-frequency dependency in chirp signals*.* The frequency sweeps considered were linear, which specifies an instantaneous frequency (*IF*) sweep given by

1 $$ IF(n)={f}_0+ Bn $$

2 $$ B=\left({f}_1-{f}_0\right)/2{n}_1 $$

In the linear sweep, *B* ensures that the desired frequency breakpoint *f*_1_ at the time *n* = *n*_1_ is maintained departing from an initial frequency *f*_0_ at the time *n* = 0. For ICG signals, it would be desirable to implement frequency variations from *f*_0_=50 bpm to *f*_1_=90 bpm in *n*_1_=10 s in order to test extremely variable conditions.

### Time-frequency distributions

High-resolution time-frequency analysis is useful for signals which are non-stationary and/or multicomponent. Quadratic TFDs (QTFD) are based on estimating the instantaneous power spectrum of the signal by using a bilinear operator [26]. The Wigner-Ville distribution (WVD) is the basic QTFD and is defined by taking the Fourier transform of an instantaneous auto-correlation function *K*_*z*_(*t*, *τ*) described in (3).

3 $$ {W}_z\left(t,f\right)={\int}_{-\infty}^{+\infty }{K}_z\left(t,\tau \right){e}^{-2 j\pi f\tau} d\tau $$

where *K*_*z*_(*t*, *τ*) is defined as

4 $$ {K}_z\left(t,\tau \right)=z\left(t+\tau /2\right){z}^{\ast}\left(t-\tau /2\right) $$

and where *z*(*t*) is the analytic associate of a real signal *x*(*t*) obtained with the Hilbert transform $z(t)=x(t)+j\mathcal{H}\left\{x(t)\right\}$. The main characteristic of the analytic signal of a real signal is that it contains no negative frequencies. Both the real and the analytic signals contain the same information but the latter has two additional beneficial effects: it halves the total bandwidth and avoid the appearance of interference terms generated by the interaction of positive and negative components in QTFDs.

The WVD provides a high-resolution representation of the signal *x*(*t*) in time and frequency, but the presence of cross-terms in multicomponent signals is deleterious for biomedical signal processing. Cross-terms can be reduced by convolving the WVD with a 2D kernel *γ*(*t*, *f*) to obtain the general formulation of QTFDs in (5).

5 $$ {\rho}_z\left(t,f\right)=\gamma \left(t,f\right)\underset{\left(t,f\right)}{\ast \ast }{W}_z\left(t,f\right) $$

The 2D kernel *γ*(*t*, *f*) reduces cross-terms but it also blurs auto-terms. Therefore, kernels need to be designed to obtain the best tradeoff between minimizing cross-terms and maintaining the auto-terms’ resolution. The general formulation of the kernels *γ*(*t*, *f*) in (5) is usually formulated in the ambiguity domain such as *g*(*ν*, *τ*), where *ν* and *τ* are Doppler and lag, as indicated in (6). This is because the convolution operation in the time-frequency domain becomes a multiplication in the ambiguity domain, which reduces its complexity.

6 $$ {\rho}_z\left(t,f\right)={\int}_{-\infty}^{\infty }{\int}_{-\infty}^{\infty }g\left(\nu, \tau \right){A}_z\left(\nu, \tau \right){e}^{j2\pi \left(\nu t- f\tau \right)} d\nu d\tau $$

where *A*_*z*_(*ν*, *τ*) is the ambiguity function of the analytic associate *z*(*t*) of the real signal *x*(*t*). Separable kernels are a special case when *g*(*ν*, *τ*) = *G*_1_(*ν*)*g*_2_(*τ*). If *G*_1_(*ν*) = 1, the kernel is Doppler independent. If *g*_2_(*τ*) = 1, the kernel is lag independent. Table 1 shows the TFDs tested along with the corresponding kernels. The spectrogram is calculated using four different windows: rectangular, Hamming, Hanning, and Bartlett.

**Table 1:** Kernels for time-frequency distribution

TFD	Kernel type	Kernel *g*(*ν*, *τ*)
WVD		1
BJD	Non-separable	sinc(2*αντ*)
S-Method	Non-separable	${A}_w\left(\nu, \tau \right)\underset{\nu }{\ast }P\left(-\nu /2\right)$
Spectrogram	Non-separable	*w*(*ν* + *τ*/2) *w*(*ν* − *τ*/2)
CWD	Non-separable	${\mathrm{e}}^{-{\upnu}^2{\uptau}^2/\sigma }$
ZAM	Non-separable	$w\left(\tau \right)\frac{a}{2\left|\tau \right|}\operatorname{sinc}\left(\frac{a}{2\left|\tau \right|}\right)$
MBD	Lag-independent	$\frac{{\left|\Gamma \left(\beta + j\pi \nu \right)\right|}^2}{\Gamma^2\left(\beta \right)}$
EMBD	Separable	$\frac{{\left|\Gamma \left(\beta + j\pi \tau \right)\right|}^2}{\Gamma^2\left(\beta \right)}\frac{{\left|\Gamma \left(\beta + j\pi \tau \right)\right|}^2}{\Gamma^2\left(\beta \right)}$

Kernels for the QTFDs [31] used in this work. The parameters *a*, *α*, *β*, and *σ* and the window length *w* define the kernel shape and are estimated taking into account the characteristics of the signal to analyze. *A*_*w*_(*ν*, *τ*) is the ambiguity domain representation of the analysis window used in the S-Method, and *P*(*ν*) is a frequency window where the width controls the cross-term suppression of the TFD

The S-Method (SM) is based on the relation of the short-time Fourier transform (STFT) and the WVD: the STFT is a linear operation and does not suffer from any cross-terms [34]. Cross-terms in TFDs appear due to the interaction of the STFTs of different signal components, which can be avoided using a window frequency function *P*(*ν*) = 0, |ν| > *L*_*P*_. By changing *L*_*P*_, a gradual transition can be obtained ranging from the spectrogram (*L*_*P*_ → 0, *P*(*ν*) = *δ*(*ν*)/2) to the WVD (*L*_*P*_ → signal length, *P*(*ν*) = 1). The best choice of *L*_*P*_ would be the value when *P*(*ν*) is wide enough to enable complete integration over the auto-terms, but narrower than the distance between the auto-terms, in order to avoid the cross-terms. Equation (7) describes how the SM is based on the STFT and the window frequency function *P*(*ν*).

7 $$ {\mathrm{SM}}_Z\left(t,f\right)=2{\int}_{-\infty}^{\infty }P\left(\nu \right){STFT}_z\left(t,f+\nu \right){STFT}_z^{\ast}\left(t,f-\nu \right) d\nu $$

### Identification of instantaneous frequency

The evaluation of different kernels by using synthetized ICG signals with known time-frequency properties can lead to the most adequate QTFD for real ICG signals. To validate this extent, the *IF* can be calculated in the QTFDs with the optimized kernels. There are several techniques to spot the different *IF*. One of the first approaches used an iterative estimate of the first moment of the spectrogram as the *IF* in order to also construct a matched spectrogram estimate. This approach is stopped when convergence between two consecutive spectrograms was reached and produces a match spectrogram concentrated along the *IF* of monocomponent signals [35]. *IF* estimation from the maxima of the TFDs has a variance and bias highly influenced by the lag window length. Therefore, Stanković and Katkovnik [36, 37] proposed an adaptive algorithm for determining the optimal lag width based on the intersection of upper and lower confidence intervals of the *IF* estimates for each time instant. The *IF* estimation methods of multicomponent signals are highly dependent on the selected TFR and presence of cross-terms which could be mistaken for the *IF* estimate. A simple approach is to use time-frequency filtering methods to retrieve individual *IF*s [26]. Other approaches require extending algorithms for estimation of monocomponent *IF* to the case of signals with various *IF* [38]. In general, concrete properties are required for the TFDs when detecting the *IF* of multicomponent signals: high time-frequency resolution and efficient suppression of cross-terms; direct amplitude estimation for the individual components and a function of the variance of the *IF* estimate continuously decreasing as the lag window length increases while the bias is continuously increasing. TFDs such as the MBD satisfy these requirements, and the work by Stanković and Katkovnik in [36, 37] can also be extended for the case of multicomponent signals.

This work proposes an algorithm to search the *IF* without setting the total number of existing IFs and their approximate frequencies. The steps are as follows:

1. Find a number *N* of local maximum peaks for every time instant in the TFD higher than the number of peaks expected.
2. Group these peaks so that they form smooth lines along time. This is the expected behavior since the synthetic TFDs are created as a Fourier series of tones whose frequency varies in a determined manner along time.

It is important to note that the first and last 10% of time of the TFD is not to be taken into consideration in order to detect the *IF*, since the starting and final instants of the TFD are blurry and can introduce errors in calculations.

### Selection of TFDs

Choosing the best kernel for a signal requires a concrete strategy. In some studies, the kernel is selected in a visual way. However, this method is highly unpredictable and biased and therefore quantification methods have been proposed. Since the characteristics of the synthetic ICG signal to analyze are well known, it is possible to quantify which kernel produces the QTFD with characteristics most similar to those of the ICG signal. Boashash and Sucic [28] proposed to measure the difference between the instantaneous frequency $\widehat{IF}(n)$ that a QTFD produced and the theoretical instantaneous frequency *IF*(*n*). For this purpose, two statistical measures are used in order to measure these differences: the mean square error (*MSE*) in (8) and the percentile root mean square difference (*PRD*) in (9).

8 $$ MSE=\frac{\sum_{n=1}^N{\left[ IF(n)-\widehat{IF}(n)\right]}^2\ }{\left(n-1\right)} $$

9 $$ PRD=\frac{\sqrt{\sum_{n=1}^N{\left[ IF(n)-\widehat{IF}(n)\right]}^2\ }}{\sqrt{\sum_{n=1}^N{\left[ IF(n)\right]}^2\ }\ } $$

where *n* is the time instant.

Furthermore, Boashash and Sucic [28, 29, 39] also proposed a method to measure the performance of a QTFD with an objective quantitative criterion expressed in (10).

10 $$ P=1-\frac{1}{3N}\sum \limits_{n=1}^N\left[\frac{A_S(n)}{A_M(n)}+\frac{1}{2}\frac{A_x(n)}{A_M(n)}+\frac{E(n)}{\Delta f(n)}\ \right] $$

where for a pair of signal components at time *n* (in the total interval of *N* time instants), *A*_*M*_ is the average of the component main lobe amplitudes, *A*_*s*_ is the average of the component sidelobes, *A*_*X*_ is the in-between component cross-term amplitude, *E* is the average of the component main lobe bandwidths, and Δ*f* is the frequency separation between the *IF* components. A low *P* indicates poor performance while values close to one indicate good performance. Both performance *P* and the different statistical measures are used to select the best TFD and its parameters.

The performance *P* calculation has undergone some changes to take into account that the tones in a synthetized ICG signal will not have the same amplitude. Equation (11) shows the adaptation of the performance for a two-tone synthetized ICG signal.

11 $$ P=1-\frac{1}{3N}\sum \limits_{n=1}^N\left[\frac{1}{2}\left|\frac{A_{s1}(n)}{A_1(n)}\right|+\frac{1}{2}\left|\frac{A_{s2}(n)}{A_2(n)}\right|+\left|\frac{A_x(n)}{A_1(n)+{A}_2(n)}\right|+\left(1-D(n)\right)\right] $$

where, *A*_*s*1_(*n*) and *A*_*s*2_(*n*) are the sidelobe amplitudes of tones 1 and 2, *A*_1_(*n*) and *A*_2_(*n*) are the tone amplitudes of tones 1 and 2, *A*_*x*_(*n*) is the amplitude of the cross-term between both tones.

12 $$ D(n)=\left({V}_1(n)/2+{V}_2(n)/2\right)/\left({f}_2(n)-{f}_1(n)\right) $$

where *V*_1_(*n*) and *V*_2_(*n*) are the bandwidths, and the instantaneous frequencies of the first and second tones are *f*_1_(*n*) and *f*_2_(*n*), respectively.

A new approach has also been used in order to validate the performance of the studied distributions. Taking into account that the WVD of a linear frequency-modulated signal gives an unbiased estimate of the *IF* of such frequency, the performance of a TFD can also be studied as the likelihood between such TFD and the addition of the WVDs of every single tone in a test signal such as the synthetic ICG signal. This likelihood can be quantified by means of the cross-correlation (CC) between the studied TFD of the synthetized ICG signal and the addition of the WVDs of every single tone in the synthetized ICG signal.

Finally, the previously mentioned measurements (*P*, *MSE*, *PRD*, and *CC*) have been applied to synthetic ICG signals with and without noise. To test the behavior of the different TFDs, noise tests have been conducted by corrupting the synthetic ICG signals with white noise in signal-to-noise ratios (SNR) decreasing from 20 to − 5 dB. According to the literature [26, 40–42], the sources of error in the estimation when detecting the *IF*s by using the TFD maximal positions are the estimate bias, the random deviation of the maxima in the auto-term due to small noise, and the large random deviations due to false detection of maximal points outside the auto-terms. In [41], an adaptive *IF* estimator with a time-varying and data-driven window length is presented, and the results are similar to the quality obtained if the *IF* information was known in advance. The work in [42] shows that the estimator of the polynomial WVD for signals with additive white Gaussian noise can be improved by the adequate selection of the kernel coefficients in the distribution. These works are expanded in [40] for the case of high noise, and it explains that the crucial parameter is the ratio of auto-term magnitude and the standard deviation of the distribution.

### Testing of the selected TFDs

Once the initial synthetic signals have been designed and the optimized TFDs have been selected, these results have been applied to the complete database of 15 patients. Synthetic ICG signals have been designed using ICG patterns extracted from the real ICG signals in every patient. The synthetic ICG signals have been used to test the performance *P*, cross-correlation, and IF errors for the optimized TFDs.

## Results

### Synthetic ICG signals

**Fig. 1:**
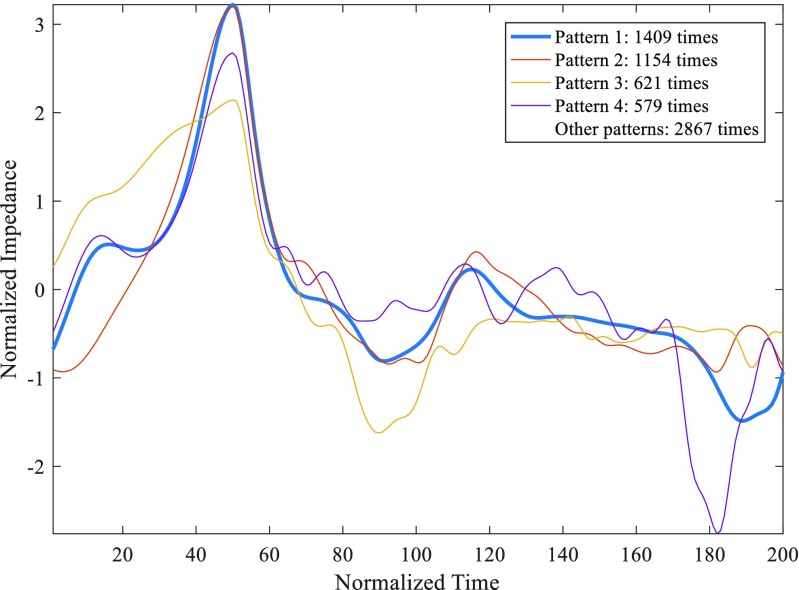
Five most frequent patterns in an ICG recording calculated with a correlation threshold of 0.85

In total, 7544 beats were analyzed for the first patient. The correlation threshold of 0.85 produced 92 different patterns with at least 4 appearances, but 8 patterns covered more than 60% of beats. Figure 1 shows the most typical patterns for this correlation threshold.

A correlation threshold of 0.90 was also studied. The ICG patterns obtained using either threshold were very similar. However, the number of patterns with at least 100 repetitions differs between both thresholds: it was higher with the *th* = 0.90 (11 patterns) compared to 10 relevant patterns obtained with a *th* = 0.85. Moreover, the main pattern was appeared 699 times in the case of the *th* = 0.90, while in the case of the *th* = 0.85, the same pattern was repeated 1154 times.

**Fig. 2:**
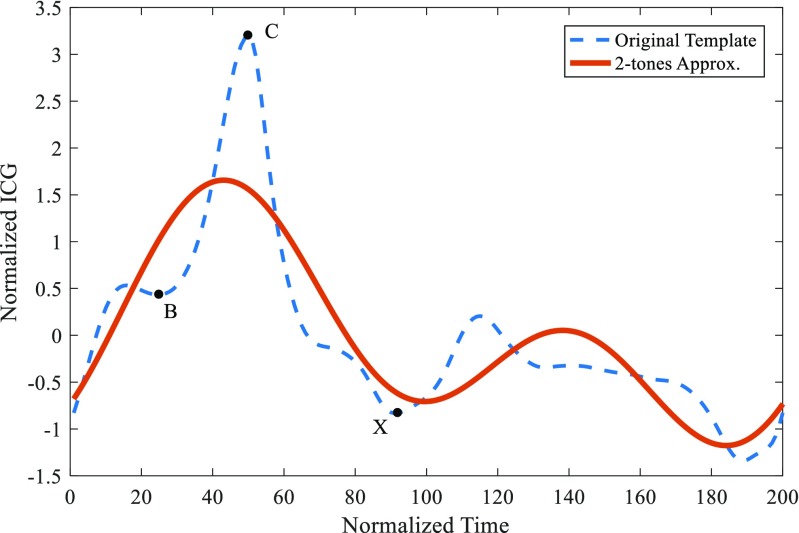
Fourier model of the ICG pattern: original ICG template (dashed blue) and an approximation of two tones of the Fourier model (red). Characteristic points B, C, and X are marked on the ICG curve

The most repetitive pattern with *th* = 0.85 was selected as the template for the Fourier modeling. However, it was necessary to slightly modify the endpoints of the signal in a smooth way so that the beginning and the end of the templates happen to meet at the same point without creating noticeable transitions when concatenating several templates together to form a longer signal. All templates have been normalized to zero mean and adjusted to a length *N*_*p*_ = 200 samples. An approximation of two tones has been used for the Fourier series with a fixed frequency. The results of the model can be observed in Fig. 2. Characteristic points B, C, and X are marked on the ICG curve: the point B coincides with the opening of the aortic valve; the point C corresponds to the peak of the ICG signal and it coincides with the ventricular contraction; and the point X corresponds to the closure of the aortic valve [43].

**Fig. 3:**
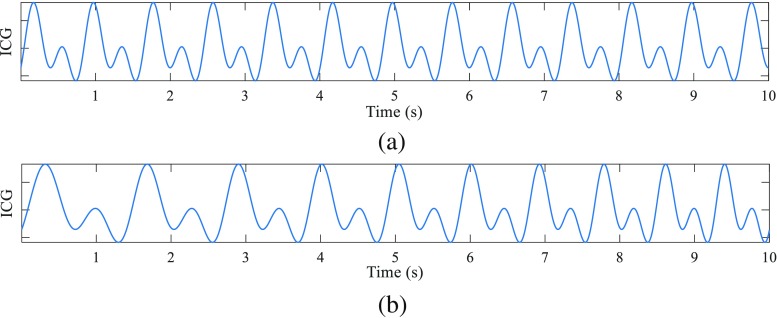
ICG signals with a constant *IF* (**a**) and a linear frequency variation (**b**) synthetized with two tones

The previous modeled Fourier approximation has been modified in order to include a linear frequency variation. Figure 3 presents a synthetized ICG with a constant *IF* and a synthetized ICG signal which frequency changes in a linear fashion from *f*_0_=50 bpm to *f*_1_=90 bpm in 10 s.

**Fig. 4:**
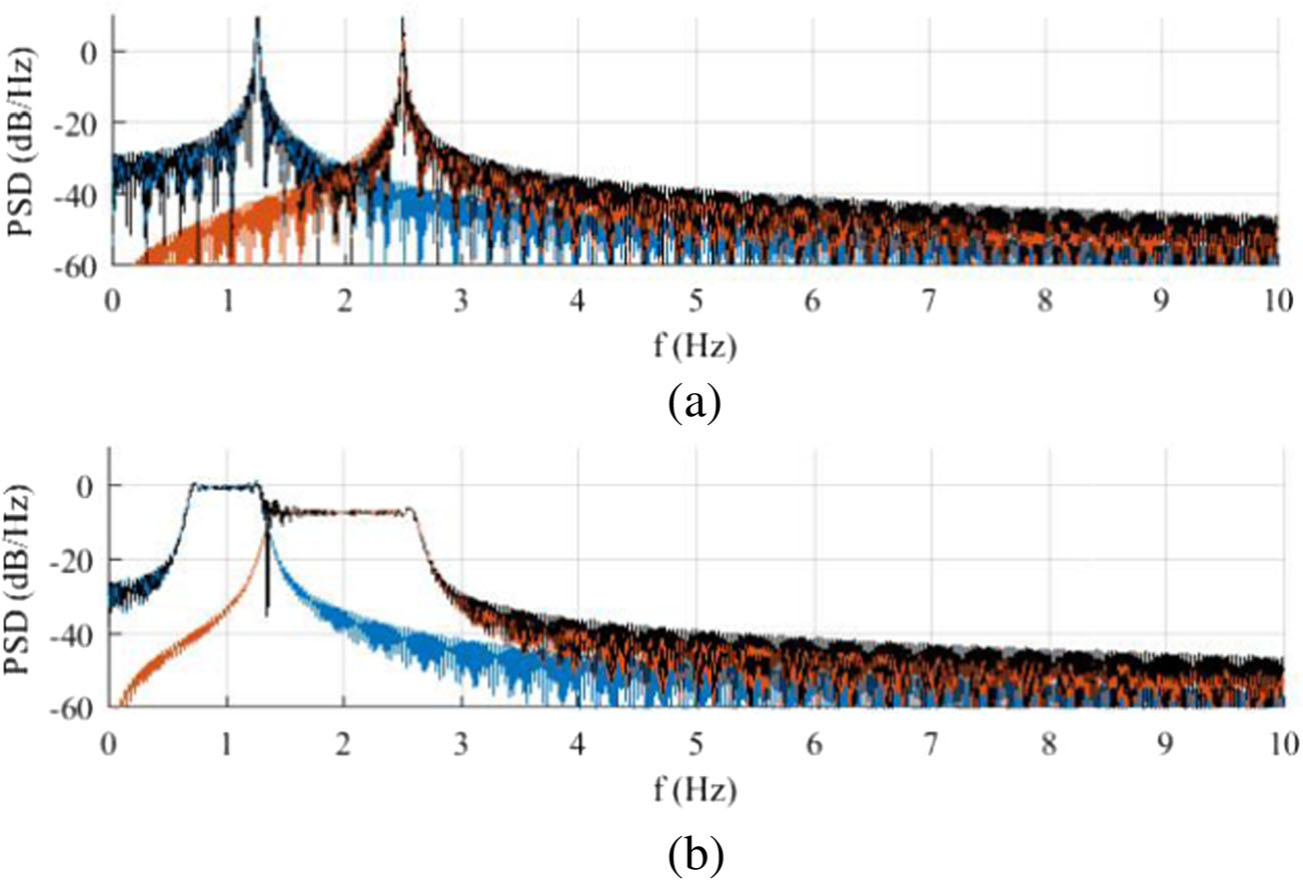
Periodograms of the synthetized ICG signals with no frequency variation (**a**) and with a linear frequency variation (**b**). Each instantaneous frequency is plotted individually (in color) and the total resulting spectra are also included (in black)

The frequency response for the Fourier model of the ICG pattern and for the linearly variable time-frequency ICG in Fig. 4 is expected to have a similar frequency response with some important differences in the shape of the frequency spectrum. The frequency response of the constant-frequency ICG presents a set of periodically distributed peaks corresponding to each of the Fourier terms, as shown in Fig. 4a. For the case of the two-tone synthesized ICG, the linear variation in the signal instantaneous frequencies makes both frequency peaks *IF*_1_ and *IF*_2_ wider (see Fig. 4b), which causes them to interfere with each other as the resulting spectrum shows (in black).

### Performance of TFDs of synthetic ICG signals

To test the performance of different TFDs, the previous synthetized two-tone ICG signal with a linear frequency variation has been used. The performance has been calculated for each type of TFD kernel with the different characteristic parameters for each type of kernel. For each TFD, the values of the parameters for the best performance have been obtained through an optimization procedure to find the combination of parameters for which the performance *P* is maximal according to (11).

**Fig. 5:**
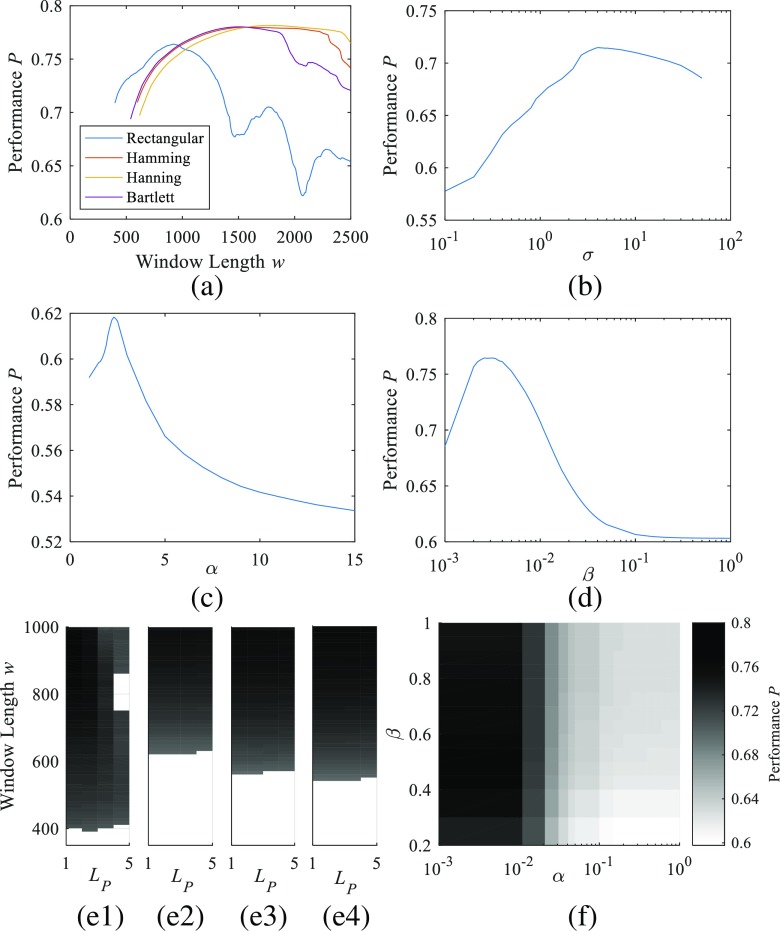
Performance optimization results: the resulting performance *P* of the spectrograms (**a**), ZAM distribution (**b**), CWD (**c**), MBD (**d**), SM (**e**), and EMBD (**f**) for varying parameters is plotted. The SM is optimized for a rectangular (e1), Hamming (e2), Hanning (e3), and Bartlett (e4) windows

The results of the optimization process are plotted in Fig. 5, and the specifications are included in Table 2. Figure 5 shows how the performance changes for each type of TFD depending on the values of several parameters such as window length *w* for the spectrogram (Fig. 5a), *σ* for the CWD (Fig. 5b), *a* for the ZAM distribution (Fig. 5c), *β* for the MBD (Fig. 5d), *L*_*P*_ and window length *w* for the SM for all window types (Fig. 5e), and *α* and *β* for the EMBD (Fig. 5f). As the figure shows, the highest *P* performances are obtained for the spectrograms (except when the rectangular window is used) and the EMBD, and the lowest *P* performances are for the CWD. The S-Method provides results very similar to those offered by the spectrogram for all cases although slightly inferior. The blank spaces in Fig. 5e represent the combination of values for which the performance calculation algorithm has not been able to identify the *IF* tones. Table 2 presents the values for these parameters for the best performance distributions, where the maximum performances are for the spectrogram when the Hamming (*P =* 0.781), Hanning, and Bartlett (*P =* 0.780) windows are used and for the EMBD (*P =* 0.778).

**Table 2:** TFD optimization results

	Parameters			MSE (%)		PRD (%)	
		*P*	*CC*	*IF*_1_	*IF*_2_	*IF*_1_	*IF*_2_
WVD		0.698	–	0.013	0.029	0.952	0.727
BJD		0.698	0.557	0.117	0.286	2.907	2.274
SM	Rect. *w* = 929, *L*_*P*_=1	0.769	0.588	0.000	0.000	1.092	0.902
	Ham. *w* = 999, *L*_*P*_=2	0.764	0.492	0.000	0.000	0.763	0.409
	Han. *w* = 999, *L*_*P*_=1	0.757	0.471	0.000	0.000	0.775	0.424
	Bart. *w* = 999, *L*_*P*_=2	0.765	0.498	0.000	0.000	0.804	0.461
Spect.	Rect. *w* = 919	0.764	0.506	0.019	0.060	1.176	1.043
	Ham. *w* = 1569	0.781	0.495	0.048	0.139	1.870	1.584
	Han. *w* = 1779	0.780	0.501	0.020	0.066	1.194	1.092
	Bart. *w* = 999	0.780	0.448	0.009	0.021	0.795	0.617
CWD	*σ* = 4.12	0.715	0.600	0.077	0.149	2.359	1.638
ZAM	*a* = 2.3	0.618	0.603	0.023	0.086	1.290	1.248
MBD	*β* = 0.0026	0.765	0.686	0.072	0.107	2.272	1.392
EMBD	*α* = 0.002, *β* = 0.988	0.778	0.577	0.077	0.114	2.362	1.432

Best performance *P* and correlation *CC* of TFDs with their parameter values; *MSE* and *PRD* for the resulting and theoretical IFs for the first (*IF*_1_) and second (*IF*_2_) tones

In addition, the *CC* correlation between the studied TFDs of the synthetized ICG real-based signal, and the addition of the WVDs of the two single tone in the synthetized ICG signal is included in the same table. The *CC* correlation is best for the MBD and worst for some of the spectrograms and S-Method reaching even less than 0.5. Finally, a comparison between the expected and the resulting instantaneous frequencies has been performed using (8) and (9), whose results are also included in Table 2.

**Fig. 6:**
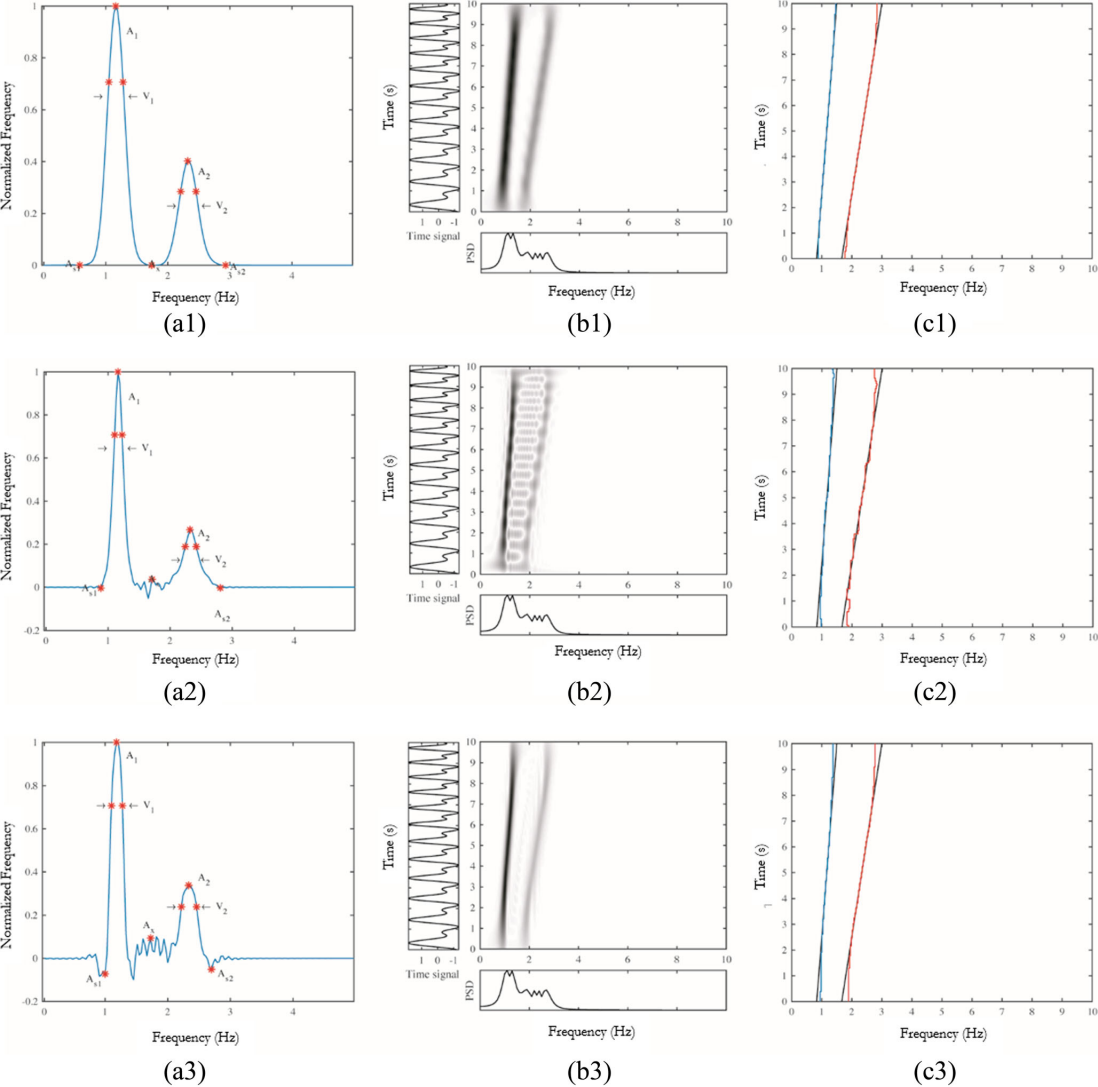
**a** A time slice at *t* = 5 s of the TFD with the characteristic points ${A}_{s_1},{A}_x,{A}_2,{A}_{s2}$, and frequency bands *V*_1_ and *V*_2_ in red of the two tones and the cross tones for the calculation of the performance *P*. **b** The resulting optimized TFD. **c** Location of the resulting first (in blue) and second (in red) IFs, *IF*_1_, and *IF*_2_ against the theoretical results (in black), for each type of analyzed TFD: spectrogram with a Bartlett window (1), CWD (2), and EMBD (3)

Table 3 includes all the numerical values of the characteristic points for the central slice, corresponding to the instant 5 s. Figure 6a contains the plot of the time slice at *t* = 5 s with the characteristic points used to calculate the performance *P* according to (10). Figure 6b shows the resulting TFDs for the kernel spectrogram with Hanning window, CWD, and EMBD, and Fig. 6c shows the theoretical IFs and the resulting IFs located on the TFDs. Some features of these kernels can be easily seen in Fig. 6. The non-negativity characteristic of the spectrogram provides zero-valued cross-term and secondary term amplitudes easy to locate.

**Table 3:** Slice optimization results

		Characteristic points in TF slice							
	*P*_*t* = 5 *s*_	*A*_*s*1_	*IF*_1_	*V*_1_	*A*_*x*_	*V*_2_	*IF*_2_	*A*_2_	*A*_*s*2_
WVD	0.668	− 0.229	1.19	39.4	0.358	46.4	2.35	0.420	− 0.076
BJD	0.522	− 0.172	1.22	198.4	0.074	247.8	2.29	0.374	− 0.103
SM Rect.	0.770	0.028	1.19	56.1	− 0.003	59.7	2.35	0.384	0.022
SM Ham.	0.763	0.000	1.19	75.8	0.000	82.7	2.35	0.395	0.000
SM Han.	0.754	0.000	1.19	85.6	0.000	87.5	2.35	0.420	0.000
SM Bart.	0.767	0.000	1.19	74.5	0.000	80.0	2.35	0.408	0.000
Spect.Rect.	0.757	0.022	1.19	166.0	0.030	178.8	2.38	0.346	0.059
Spect. Ham.	0.776	0.000	1.19	159.3	0.000	286.9	2.35	0.263	0.000
Spect. Han.	0.781	0.000	1.19	174.6	0.000	217.3	2.35	0.333	0.000
Spect. Bart.	0.768	0.000	1.19	228.0	0.000	246.9	2.35	0.402	0.000
CWD	0.716	− 0.072	1.19	176.1	0.094	243.8	2.35	0.338	− 0.051
ZAM	0.605	− 0.456	1.19	86.1	− 0.109	109.3	2.35	0.285	− 0.167
MBD	0.753	− 0.014	1.19	65.00	0.173	83.0	2.35	0.244	0.016
EMBD	0.782	0.004	1.19	119.6	0.037	177.0	2.35	0.267	− 0.003

Amplitudes ${A_{s_1}}_1,{A}_x,{A}_2,{A}_{s2}$ and frequency bands *V*_1_ and *V*_2_ (in mHz) of the *IF*_1_ and *IF*_2_ (in Hz) for the calculation of the instant performance *P*_*t* = 5*s*_. Amplitude *A*_1_ is always the unit

However, in Fig. 6, the frequency width of the IFs is larger for the spectrogram than for other kernels such as the MBD and the WVD. It is also noticeable how the spectrogram and the EMBD kernels provide a softer TFD with fewer cross-terms between the two frequencies, which are prominent in the case of the CWD. A large number of undulations appear in addition to the two main ridges which represent the sum of the two frequency-modulated signals. In the basic WVD, the cross-terms are located midway between the interacting components, oscillate proportionally to the distance between the auto-terms and in a direction orthogonal to the line connecting these auto-terms. Quadratic TFDs in which cross-terms are attenuated relative to the auto-terms in often named a reduced interference distribution (RID), and it is a well-studied topic in the literature [26, 44]. In general, since auto-terms in the *(t,f)* plane are usually smooth, their corresponding version in the *(ν, τ)* plane tends to be concentrated in the origin. On the contrary, Fig. 6 shows that cross-terms tend to be oscillatory in the *(t,f)* plane which lead to terms far away from the origin in the *(ν, τ)* plane. Choosing the right kernel in the *(ν, τ)* plane can filter out information far from the center and can thus attenuate cross-terms. In addition, the starting and final seconds of the TFD seem fuzzier than the rest of the TFD. This fact is to be taken into account when calculating the IFs of the distributions.

**Fig. 7:**
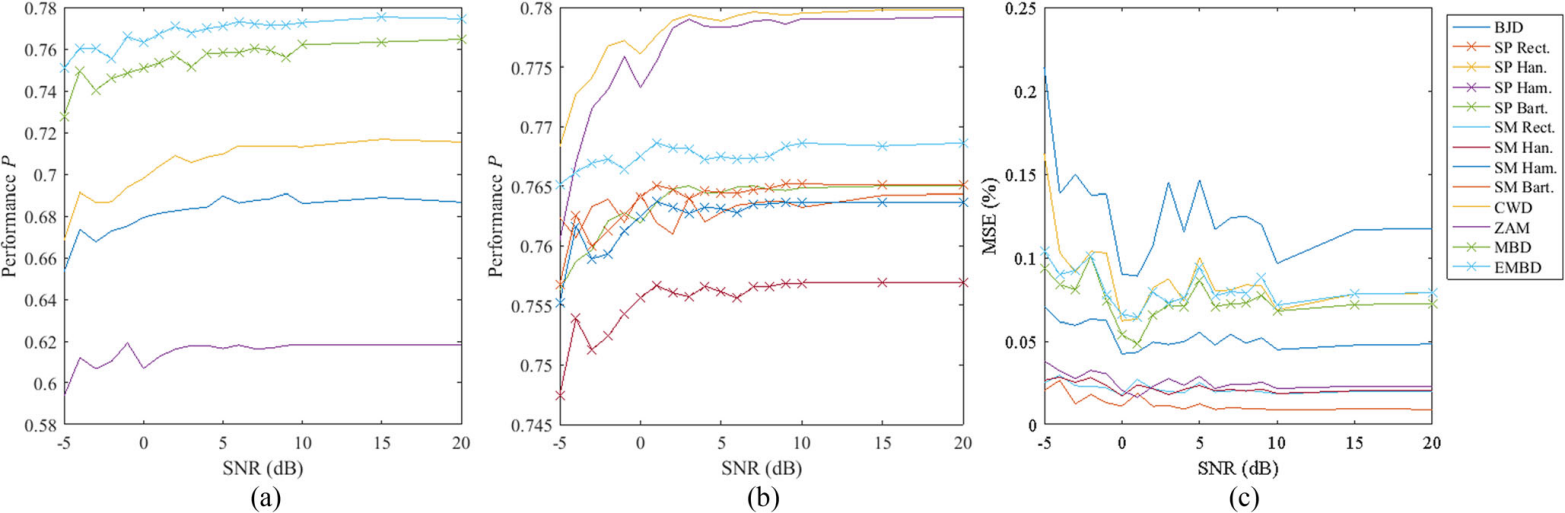
Results to noise tests. **a** and **b** show the performance *P* of several TFDs to different SNR rates and **c** shows the root mean square error (*MSE*) in the detection of the first instantaneous frequency

Finally, the analysis of the robustness of the different kernels in relation to the signal-to-noise ratio (SNR) is summarized in Fig. 7. Noise in TFDs has been previously analyzed in depth, and the bias and variance for different types of additive and multiplicative noise have been determined [45]. The study of the additive Gaussian noise influence on TFDs has led to the design of robust TF distribution using the robust minimax Huber M-estimates [46, 47].

In this study, all kernels except ZAM, CWD, and BJD present similar performance *P* between 0.76 and 0.78 when SNR = 20 dB, and such performance decreases at a similar speed for all cases until reaching a performance *P* between 0.73 and 0.76 when SNR = − 5 dB although the case at which the *P* decreases is slightly higher for EMBD and MBD. Regarding Fig. 7c, the *MSE* values are not similar for all kernels and vary from 0.01 to 0.12 for SNR = 20 dB and from 0.02 to 0.22 for SNR = − 5 dB. However, the evolution of the decrease in *MSE* with decreasing SNR is similar for all kernels, and the *MSE* values remain constant after 10 dB.

### Testing of the selected TFDs

**Fig. 8:**
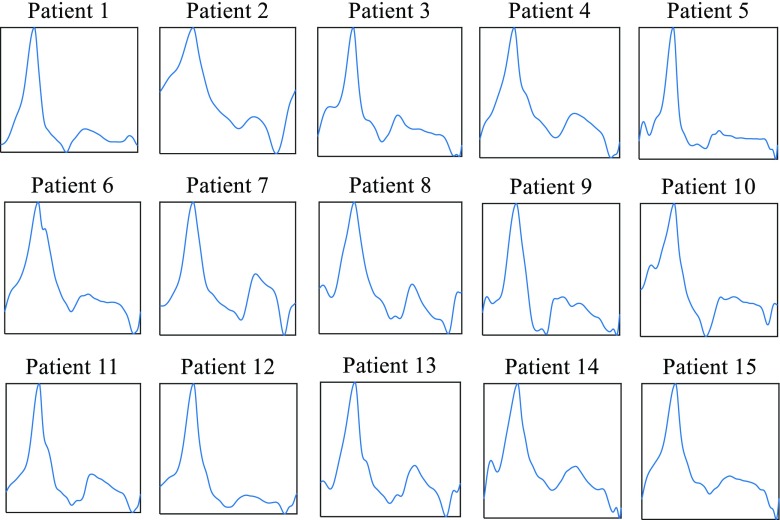
Main ICG pattern for all patients in the data base. *X*-axis is normalized time and *Y*-axis is normalized ICG. Patterns have been normalized to the same time duration in the *X*-axes

The most frequent pattern has been extracted from every patient with a correlation coefficient of *th =* 0.85 between ICG beats, as in the case for the first synthetized ICG signal described above. All patterns are included in Fig. 8. These patterns have been used to create synthetic ICG signals with known linear time-frequency variations in order to calculate their TFDs with different kernels and test their performances. Table 4 includes the performance *P*, performance at central time *P*_*t* = 5*s*_, cross-correlation performance *CC*, and *IF MSE* and *PRD* errors. The performance *P* is best for the spectrogram when either Hanning or Hamming windows are used and for the EMBD, while the WVD and the ZAM offer the worst results. However, the WVD also offers some of the best results for the location of the instantaneous frequency.

**Table 4:** TFD results for the complete database

	Performance			MSE (%)		PRD (%)	
	*P*	*P*_*t* = 5 *s*_	*CC*	*IF*_1_	*IF*_2_	*IF*_1_	*IF*_2_
WVD	0.601 ± 0.011	0.610 ± 0.010	–	0.016 ± 0.003	0.017 ± 0.006	1.081 ± 0.099	0.542 ± 0.087
BJD	0.686 ± 0.003	0.684 ± 0.004	0.603 ± 0.027	0.095 ± 0.005	0.196 ± 0.041	2.617 ± 0.074	1.871 ± 0.181
SM Rect.	0.762 ± 0.002	0.762 ± 0.002	0.577 ± 0.004	0.027 ± 0.009	0.030 ± 0.017	1.380 ± 0.230	0.715 ± 0.168
SM Ham.	0.780 ± 0.000	0.780 ± 0.000	0.465 ± 0.002	0.008 ± 0.000	0.009 ± 0.001	0.781 ± 0.013	0.396 ± 0.016
SM Han.	0.780 ± 0.000	0.780 ± 0.001	0.483 ± 0.003	0.008 ± 0.000	0.009 ± 0.000	0.785 ± 0.014	0.415 ± 0.006
SM Bart.	0.765 ± 0.000	0.765 ± 0.000	0.490 ± 0.003	0.009 ± 0.001	0.011 ± 0.001	0.824 ± 0.033	0.446 ± 0.012
Spec. Rect.	0.710 ± 0.003	0.714 ± 0.004	0.657 ± 0.005	0.022 ± 0.009	0.046 ± 0.018	1.244 ± 0.236	0.896 ± 0.153
Spec. Ham.	0.614 ± 0.003	0.620 ± 0.001	0.634 ± 0.014	0.038 ± 0.003	0.145 ± 0.006	1.655 ± 0.067	1.616 ± 0.035
Spec. Han.	0.764 ± 0.002	0.767 ± 0.002	0.621 ± 0.028	0.013 ± 0.001	0.068 ± 0.003	0.964 ± 0.043	1.103 ± 0.028
Spec. Bart.	0.776 ± 0.001	0.782 ± 0.001	0.587 ± 0.003	0.010 ± 0.001	0.020 ± 0.001	0.832 ± 0.037	0.596 ± 0.023
CWD	0.766 ± 0.001	0.767 ± 0.001	0.640 ± 0.027	0.064 ± 0.003	0.121 ± 0.017	2.145 ± 0.052	1.476 ± 0.097
ZAM	0.764 ± 0.000	0.765 ± 0.000	0.692 ± 0.030	0.018 ± 0.001	0.073 ± 0.014	1.154 ± 0.039	1.143 ± 0.102
MBD	0.757 ± 0.000	0.758 ± 0.000	0.798 ± 0.031	0.048 ± 0.003	0.107 ± 0.006	1.863 ± 0.065	1.387 ± 0.042
EMBD	0.765 ± 0.000	0.766 ± 0.000	0.699 ± 0.026	0.054 ± 0.005	0.116 ± 0.005	1.978 ± 0.099	1.445 ± 0.031

Average of performance measures performance *P*, performance *P*_*t* = 5*s*_of the time slice at *t =* 5 s, and cross-correlation performance *CC*, and *IF* in *MSE* and *PRD* errors for all cases using several TFDs

## Discussion

In this work, the primary key finding is the proposal of an innovative methodology for choosing a suitable TFD for a real biomedical signal. On the one hand, several authors have previously addressed the complexity of selecting kernels for TFDs by using visual methods or characteristics of the signals to analyze [26]. Nevertheless, this work takes into account that such characteristics are often partially unknown, and the proposed methodology has been applied to ICG signals. On the other hand, some authors have used wavelet transforms for denoising ICG signals and for locating the characteristic points of the ICG curve [11, 12, 48, 49]. The main difference is that wavelet analysis uses some given analyzing wavelets (such as the so-called Mexican hat or Morlet wavelets) while the objective of this work was to investigate the overall time-frequency content of the ICG signals.

Taking the most characteristic pattern of a patient’s ICG signal, a signal with a pre-defined linearly variant time-frequency characteristics has been constructed and used to calculate the goodness of several TFDs. The goodness has been calculated using the geometrical characteristics of the TFD according to formulation adapted from prior publications [28, 29, 39], and the *MSE* (mean square error) and the *PRD* (percentile root mean square difference) between the expected and the theoretical IFs of the TFDs. Furthermore, a new performance measure has been introduced taking into account the fact that the *IF* (instantaneous frequency) detected in a WVD for a frequency linearly variant signal is the best approximation of the instantaneous frequency of the signal. Thus, the *CC* (cross-correlation) of several TFDs and the summation of the Wigner-Ville distributions of the input tones have been calculated.

The second key finding relates to the different TFDs analyzed. In our results, neither the spectrogram nor the newer MBD and EMBD are clearly superior to the other. According to the results in Table 2, the kernels with best *P* performances are the EMBD and the spectrogram when used with Hamming, Hanning, or Bartlett windows (approximately 0.8). The S-Method provides results similar to the spectrogram although slightly inferior. The MBD and the spectrogram with the rectangular windows also present high *P* performance, but it should be noted that the spectrogram is more sensitive to the length of the rectangular window than to the length of the rest of the windows, as it can be seen in Fig. 5a. Figure 5f also shows that the EMBD provides a stable performance *P* for a large variety of values for the parameters *α* and *β*. The correlation *CC* is best for MBD and worst for the spectrogram and the S-Method. However, this seems not to define the ability to detect the *IF* as predicted since the best *MSE* for both *IF*_1_ and *IF*_2_ is for the spectrogram with the Bartlett window. The *MSE* and *PRD* for the WVD are also very low, which is in line with the theoretical description of the WVD having a perfect *IF* when the signal tone changes in a linear fashion.

Regarding the performance *P* in the central time slice of the TFDs, the best performance *P* is such of EMBD followed by the Hanning spectrogram. It is important to note that there are almost no secondary and cross lobes. Nevertheless, the thinnest tone is detected by MBD (after the WVD for the reasons explained above). In fact, the spectrogram provides the widest tones irrespective of the window in use. This is detrimental for the location of instant tones such as the ones used in this work. Moreover, the cross-correlation performance *CC* has offered poor results for the spectrograms while the resulting *CC* is best for MBD.

In general, the robustness of the spectrogram is related to the lack of undesirable artifacts present in other TFDs since the non-linearity is introduced in the final step of the spectrogram computation (when taking the squared magnitude). Nonetheless, the spectrogram does not satisfy the instantaneous frequency criterion of the quadratic class of TFDs, and hence, it does not allow the exact extraction of the signal IFs from its dominant peaks [26].

Regarding the noise test, the results have shown that no TFD stands out for its resistance to noise. The performance of the spectrogram seems to decrease at a slower speed than the performance of other TFDs with similar performance such as MBD and EMBD. The root mean square errors between the expected *IF* and the calculated *IF* follow the same pattern for all TFDs and start to rise for SNR less than 2 dB. Finally, the tests on the whole database of patients confirm the above-mentioned discussion, since the results have been very similar, according to Table 4.

## Conclusion

This new technique sheds light on the methodological aspects of the selection of the most adequate kernel to analyze ICG signals. In this case, some traditional kernels such as BJD could be discarded due to the results of this work, and kernels such as the spectrogram with either Hanning or Hamming windows and the extended modified beta distribution are recommended. As a guideline for future research, the final choice of a time-frequency kernel must follow a thorough evaluation of the measurements described in this paper and the user’s experience on the type of signals to analyze. Advances in the time-frequency characterization of ICG signals may lead to an increase knowledge of the morphology of these signals and their use to characterize patient’s hemodynamic situation.
